# Identification of 4-methylation driven genes based prognostic signature in thyroid cancer: an integrative analysis based on the methylmix algorithm

**DOI:** 10.18632/aging.203338

**Published:** 2021-08-29

**Authors:** Zhiwei Chen, Xiaoli Liu, Fangfang Liu, Guolie Zhang, Haijian Tu, Wei Lin, Haifeng Lin

**Affiliations:** 1 Department of Gastroenterology, The Affiliated Hospital of Putian University, Putian 351100, Fujian Province, China; 2 Department of Thyroid Surgery, The Affiliated Hospital of Putian University, Putian 351100, Fujian Province, China; 3 Clinical Laboratory, The Affiliated Hospital of Putian University, Putian 351100, Fujian Province, China; 4 Department of Gastrointestinal Surgery, The Affiliated Hospital of Putian University, Putian 351100, Fujian Province, China; 5 Department of Pathology, The Affiliated Hospital of Putian University, Putian 351100, Fujian Province, China

**Keywords:** ALDOC, C14orf62, DVL1, PTPRC, TCGA

## Abstract

Thyroid cancer (TC) is known with a high rate of persistence and recurrence. We aimed to develop a prognostic signature to monitor and assess the survival of TC patients. mRNA expression and methylation data were downloaded from the TCGA database. Then, R package *methylmix* was applied to construct a mixed model was used to identify methylation-driven genes (MDGs) according to the methylation levels. Furthermore, an MDGs based prognostic signature and predictive nomogram were constructed according to the analysis of univariate and multivariate Cox regression. Totally 62 methylation-driven genes that were mainly enriched in substrate-dependent cell migration, cellular response to mechanical stimulus, et al. were found in TC tissues. aldolase C (AldoC), C14orf62, dishevelled 1 (DVL1), and protein tyrosine phosphatase receptor type C (PTPRC) were identified to be significantly related to patients' survival, and may serve as independent prognostic biomarkers for TC. Additionally, the prognostic methylation signature and a novel prognostic, predictive nomogram was established based on the methylation level of 4 MDGs. In this study, we developed a 4-MDGs based prognostic model, which might be the potential predictors for the survival rate of TC patients, and this findings might provide a novel sight for accurate monitoring and prognosis assessment.

## INTRODUCTION

Thyroid cancer (TC) is one of the most commonly diagnosed cancers, and approximately 567,000 cases were reported worldwide in 2018 [1]. Despite a relatively low mortality rate, TC persistence and recurrence are still high [2]. Multiple risk factors may lead to TC, including smoking, obesity, radiation exposure, and overweight [3]. A variety of epigenetic and genetic alterations in follicular epithelial cells are also considered to be significant for TC initiation and progression [4, 5]. Imaging modalities, such as ultrasound examination, and some tumors markers are usually utilized for detection, while fine-needle aspiration (FNA) is the standard method used for TC biopsy. However, traditional methods are limited by their subjectivity, low sensitivity, and specificity. Therefore, reliable biomarkers are urgently needed for the accurate monitoring and prognosis assessment for malignant thyroid nodules [6].

Some studies have reported the prognostic signatures of thyroid cancer using different kinds of methods. Based on survival curves, receiver-operator characteristic curves, risk score, survival status, and independent prognostic analysis, a novel 5 immune-associated genes signature for predicting the prognosis of patients with thyroid cancer was established [7]. A four miRNA signature was identified to predict overall survival of Papillary thyroid cancer patients [8]. Glucose metabolism features of thyroid cancer were believed to be the biological progression markers, and might provide clinical implications for risk stratification [9].

Epigenetic and genetic alterations are significant for the process of cancer development and progression [10]. Some genetic changes that affect phosphatidylinositol 3-kinase/AKT pathways and mitogen-activated protein kinase in TC have been reported. Additionally [11, 12], an increasing number of studies stated that epigenetic modifications, especially DNA methylation, are found in TC [13, 14]. DNA methylation is a type of the well-characterized epigenetic process. Methylation alterations of the promoter region, which mainly occur in cytosines that precede a guanine (CpG), play crucial roles, including the maintenance of chromosomal stability and regulation of gene expression and DNA recombination, in the biological process [15]. Also, The aberrant methylation of DNA sequences, including hypomethylation of oncogenes and hypermethylation of tumor-suppressor genes, have been found to act as significant events in thyroid tumorigenesis [16]. Identification of DNA methylation alterations can help clarify the redundancy and instability of the TC genome and provide risk prediction and potential therapeutic targets. Thus, studies on DNA methylation may provide novel insight for finding effective biomarkers for monitoring and prognostic assessment of TC [17].

In recent years, growing evidence has suggested that the development and progression of TC is a multi-gene, multi-factor, and multi-stage process, in which aberrant DNA methylation is one of its crucial mechanisms [18, 19]. In order to further understand the potential mechanism of DNA methylation alterations in TC, various experiments and bioinformatics analyses were performed. The Cancer Genome Atlas (TCGA) database provides open access to cancer epigenetic and genetic profiles for researchers around the world. Thus, the relevant genomic changes and data could be applied for exploring alterations caused by DNA methylation. The *Methylmix* is an algorithm that was designed for an in-depth analysis of DNA methylation in 2015 and was improved in 2018 [20, 21]. Compared with the traditional methods, the *Methylmix* combined DNA methylation data and transcriptome data, which provides readily available, accurate, and reliable results. In this study, the transcriptome, methylation, and clinical outcome data of TC patients were downloaded from the TCGA database. Then, the *Methylmix* was used to identify cancer methylation status and analyze the methylation-driven genes (MDGs). After a series of comprehensive validation experiments and bioinformatical analysis, a 4-methylation gene prognostic signature was found to effectively predict the prognosis of TC, which provides a novel insight for TC evaluation and prognosis monitoring.

## RESULTS

### Identification of MDGs in TC

**Figure 1:**
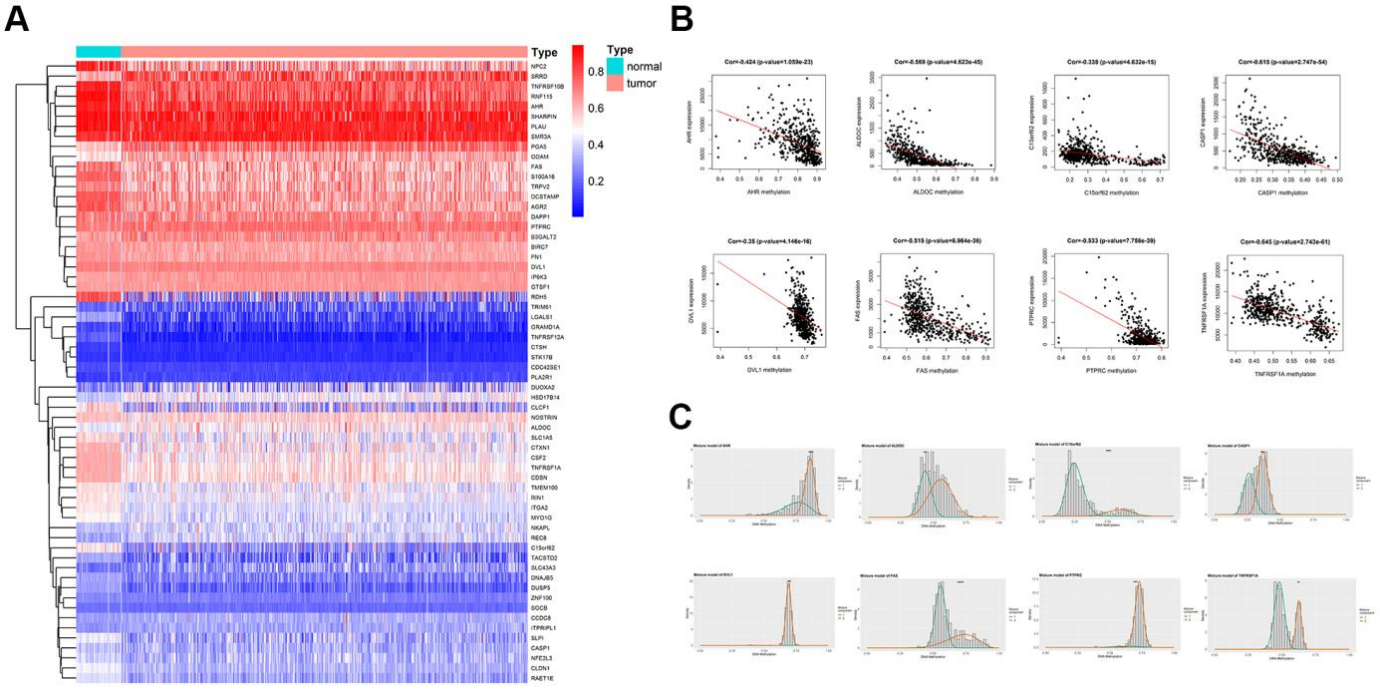
**Overview of methylation driven genes in TC.** (**A**) Heatmap of 62 methylation driven genes in TC. (**B**) Representative correlation plots of the MDGs, reflecting the correlation between expression and methylation levels of genes. (**C**) Representative distribution plots of MDGs, reflecting the distribution of methylation values.

The differentially methylated genes (DMGs) and differentially expressed genes (DEGs) were identified by using R package Limma and edge, respectively. The p-value <0.05, |logFC≥1| were used as the screening condition. Totally 486 genes were hypomethylated, and 397 genes were hypermethylated, while 1679 genes were downregulated, and 1751 genes were upregulated. Based on the R package methylmix, the DMGs and DEGs were reorganized as a normal methylated set, methylated cancer set, and gene expression cancer set. A p-value<0.05 and **|**cor**|**>0.3 were used as a standard criterion for the screening of the MDGs. Through a mixture model and Wilcoxon rank test, 62 MDGs were found. A heatmap was generated according to the methylation level of the 62 MDGs, with the R package pheatmap (Figure 1A). Also, the plots of correlation and the distribution map of the methylation degree were shown in Figure 1B, 1C. Representative pictures were selected in Figure 1, and the full results for MDGs were available in Table 1.

**Table 1:** Methylation-driven genes in thyroid cancer.

**Gene symbol**	**LogFC**	**P value**	**Adj.P value**	**Correlation**
TNFRSF12A	-1.293	7.15E-30	6.37E-28	-0.345880771
CLDN1	-0.39085	6.72E-28	5.98E-26	-0.646378569
RAET1E	-0.40497	1.25E-26	1.11E-24	-0.595721852
RDH5	-1.12623	1.98E-24	1.76E-22	-0.634947221
ITGA2	-0.31327	2.69E-23	2.40E-21	-0.436472079
PGA5	0.325305	6.34E-23	5.64E-21	-0.462713424
AGR2	-0.24707	2.93E-22	2.61E-20	-0.423001137
LGALS1	-0.95989	1.24E-21	1.11E-19	-0.365578312
TNFRSF10B	-0.18754	2.49E-21	2.22E-19	-0.428823826
CTSH	-0.37149	2.66E-21	2.37E-19	-0.320484242
DUSP5	-0.56389	1.07E-20	9.52E-19	-0.552547133
C15orf62	-0.72257	2.24E-20	2.00E-18	-0.337596483
ODAM	0.289174	5.35E-20	4.76E-18	-0.477121602
NPC2	-0.38227	5.55E-20	4.94E-18	-0.498572189
CSF2	-0.33614	6.66E-20	5.93E-18	-0.479273032
DCSTAMP	-0.24137	6.31E-19	5.62E-17	-0.721570243
HSD17B14	0.35519	8.08E-19	7.19E-17	-0.354144306
SLPI	-0.45521	9.77E-19	8.69E-17	-0.316453977
SRRD	0.315083	1.05E-18	9.39E-17	-0.458191351
RNF115	-0.23051	3.86E-18	3.44E-16	-0.412335706
TNFRSF1A	-0.25335	4.36E-18	3.88E-16	-0.644852304
MYO1G	-0.27485	1.12E-17	1.00E-15	-0.455049343
CTXN1	-0.31385	2.58E-17	2.30E-15	-0.554055233
CDSN	-0.21699	4.65E-16	4.14E-14	-0.487963678
REC8	0.357252	4.75E-16	4.23E-14	-0.422275448
PLAU	-0.07261	1.65E-15	1.47E-13	-0.424509492
S100A16	-0.27573	2.95E-15	2.63E-13	-0.483665008
RIN1	-0.2118	2.95E-13	2.63E-11	-0.566552552
CLCF1	-0.52872	4.17E-13	3.71E-11	-0.450370881
CDC42SE1	-0.16058	3.53E-12	3.14E-10	-0.330411639
IP6K3	0.057686	3.80E-12	3.38E-10	-0.439770241
FAS	-0.20232	9.38E-12	8.35E-10	-0.514968025
DNAJB5	-0.30803	2.44E-11	2.18E-09	-0.44279819
TMEM100	-0.23471	3.97E-11	3.53E-09	-0.520397785
AHR	-0.09629	4.18E-11	3.72E-09	-0.424275388
SLC1A5	-0.26678	5.12E-11	4.56E-09	-0.548239819
ZNF100	-0.11242	7.13E-11	6.35E-09	-0.319994708
FN1	-0.06458	2.84E-10	2.53E-08	-0.538405427
STK17B	-0.23421	5.21E-10	4.64E-08	-0.384776697
PLA2R1	0.186105	6.66E-10	5.93E-08	-0.400548646
ITPRIPL1	0.178554	1.35E-09	1.20E-07	-0.411437091
ALDOC	0.219977	2.18E-09	1.94E-07	-0.568851978
SGCB	-0.0891	1.57E-08	1.40E-06	-0.336380891
B3GALT2	0.084146	2.05E-08	1.82E-06	-0.618203613
CCDC8	0.157095	1.04E-07	9.22E-06	-0.615784507
TRPV2	-0.16571	1.14E-07	1.01E-05	-0.337809304
SHARPIN	-0.07134	4.31E-07	3.83E-05	-0.392771059
BIRC7	-0.05422	5.21E-07	4.64E-05	-0.527657422
NKAPL	0.182314	8.63E-07	7.68E-05	-0.458131626
NFE2L3	-0.18721	9.14E-07	8.13E-05	-0.705485784
TACSTD2	-0.38532	1.28E-06	0.000114	-0.724847508
SLC43A3	0.371821	1.36E-06	0.000121	-0.623501436
DUOXA2	0.490037	1.73E-06	0.000154	-0.384749995
DVL1	-0.01633	1.73E-06	0.000154	-0.349618498
SMR3A	0.039536	1.09E-05	0.000967	-0.373153773
CASP1	-0.1587	1.45E-05	0.001289	-0.614554241
GTSF1	0.026805	2.72E-05	0.002421	-0.467370091
GRAMD1A	-0.361	6.69E-05	0.005956	-0.435419813
NOSTRIN	-0.07267	0.000156	0.013913	-0.522905715
PTPRC	0.046445	0.000304	0.027035	-0.533390616
DAPP1	-0.04872	0.000378	0.033663	-0.769055711
TRIM61	-0.02568	0.000472	0.041989	-0.542713508

### Functional enrichment analysis of the MDGs

**Figure 2:**
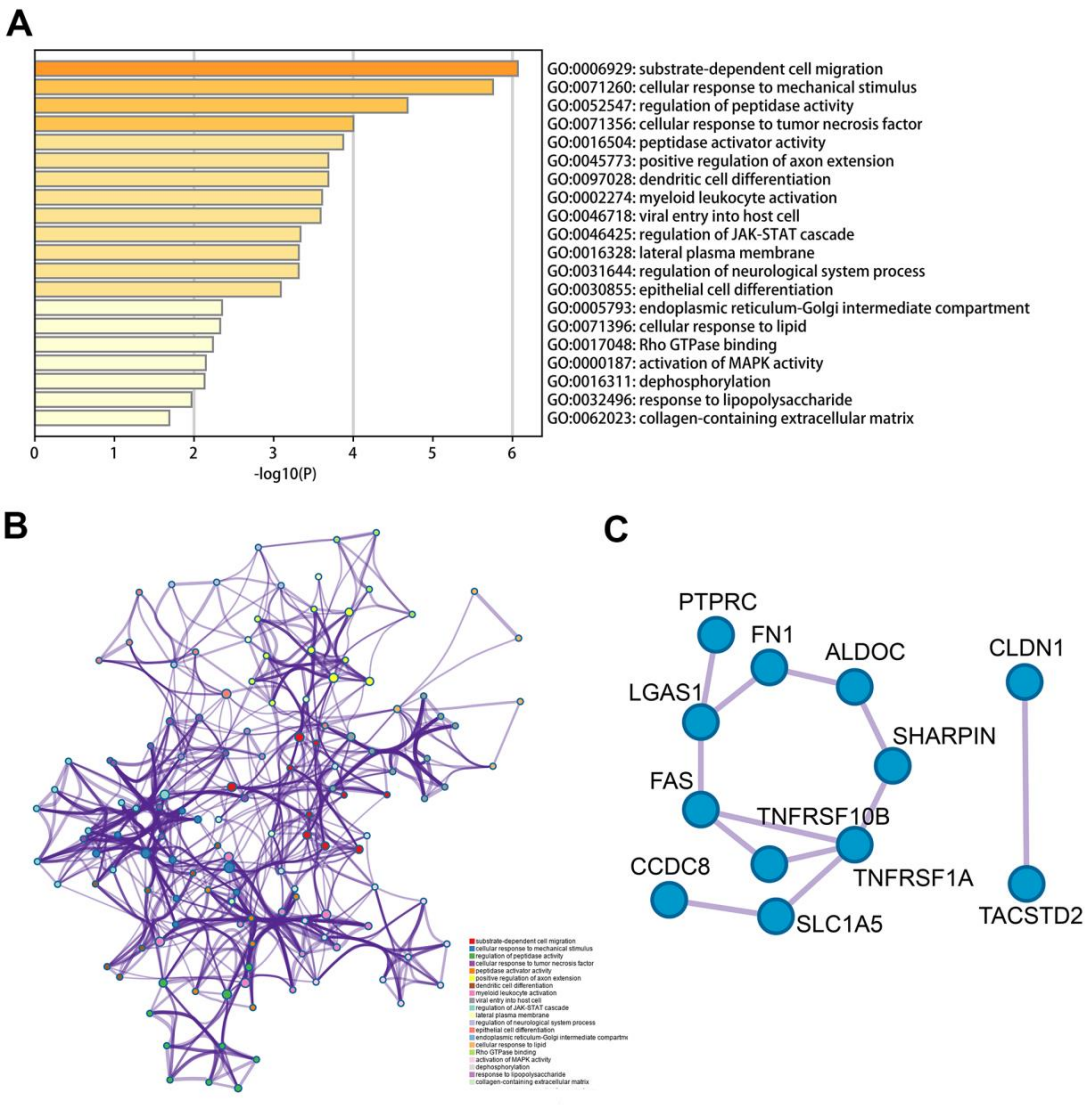
**Functional analysis of 62 MDGs based on the metascape.** (**A**) Bar graph of enriched terms across input gene lists, colored by p-values. (**B**) The network of enriched terms colored by cluster-ID, where nodes that the same cluster-ID are typically close to each other. (**C**) Protein-protein interaction network identified in the MDGs.

We conducted functional enrichment and pathway analysis using Metascape online on the base of 62 MDGs previously obtained. As shown in Figure 2A, the methylation-driven genes were mainly enriched in substrate-dependent cell migration, cellular response to mechanical stimulus, cell-substrate adhesion, cellular response to tumor necrosis factor, dendritic cell differentiation. Besides, the methylation gene set was mainly enriched in the regulation of JAK-STAT, activation of MAPK activity pathways (Figure 2B). Usually, hypermethylation inhibits transcription, while hypomethylation increased the expression of oncogenes. The results of the functional analysis revealed the potential mechanisms of MDGs.

Furthermore, to investigate the hub genes which play significant roles in TC, we constructed a PPI network and MCODE to identify the critical units. As shown in Figure 2C, module 1 included 11 edges and 12 nodes and involved ALDOC, PTPRC, FAS, etc. Meanwhile, module 2 included 1 edge and 2 nodes, and involved CLDN1, TACSTD2.

### Construction and validation of the MDGs based on predictive model

**Figure 3:**
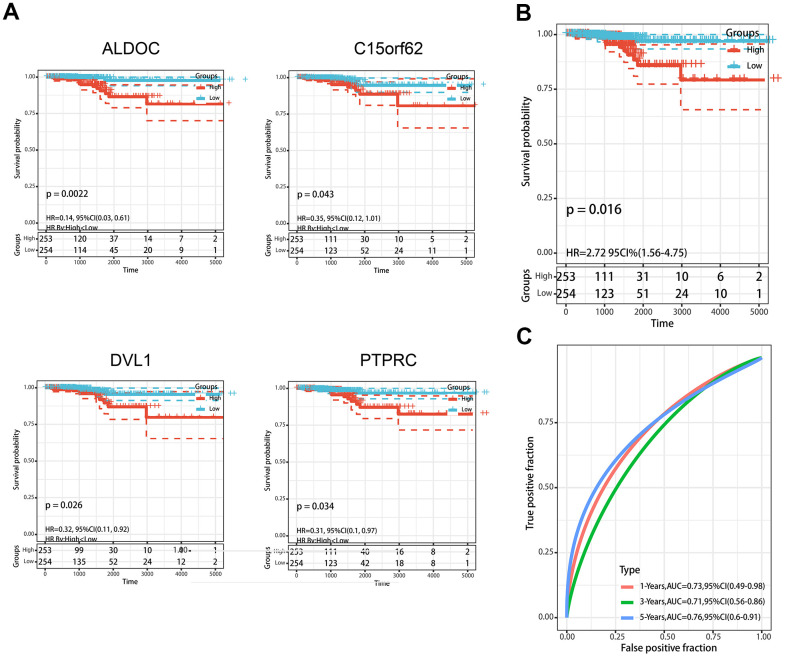
**Construction of MDGs based prognostic signature.** (**A**) Kaplan-Meier plots of ALDOC, C14orf62, DVL1, PTPRC. (**B**) The Kaplan-Meier plot of the prognostic signature based on the median of the risk score. (**C**) The ROC curve for assessing the prediction value of the signature.

Normalized methylation data with complete clinical information were obtained from the TCGA database. To enhance the reliability and accuracy of the predictive model based on MDGs, we treated 82 patients from our center as a validation set. Univariate cox regression analysis was first performed to identify the genes that were significantly associated with the prognosis from the 62 MDGs previously obtained. A total of 4 MDGs were then found to be prognosis-related according to their methylation β value (Figure 3A). Subsequently, Multivariate Cox regression analysis was conducted, and 4 MDGs (ALDOC, C14orf62, DVL1, PTPRC) were eventually selected to construct a predictive model. A risk score of each patient was generated as following: (3.23)× β value (ALDOC) + (2.98)× β value (C14orf62)+ (-8.96)× β value (DVL1) + (18.23)× β value (PTPRC). Then, the patients were divided into a high-risk group (n= 253) and a low-risk group (n= 254) according to the risk scores. The median survival time of patients with high risk was significantly higher than that of the patients with low risk (Figure 3B). Subsequently, the ROC curve was performed to assess the efficiency prognosis prediction of this model, and we found the AUC of the ROC curve of the predictive model exceeded that of the individual genes (Figure 3C).

**Figure 4:**
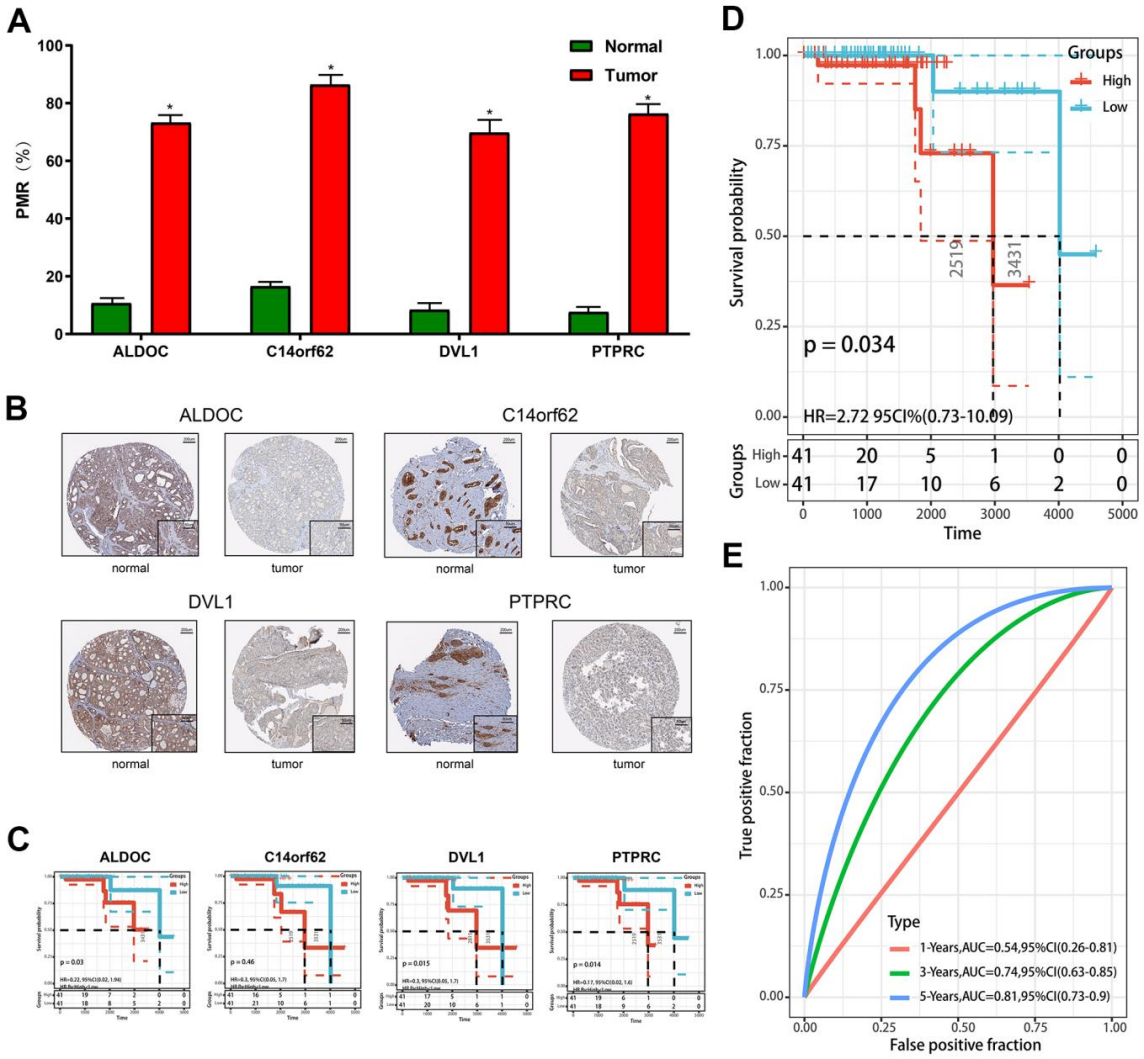
**Validation of constructed prognostic signature.** (**A**) The methylation level was detected by qMSP. (**B**) The protein level was detected by IHC. (**C**) The Kaplan-Meier plots of the ALDOC, C14orf62, PTPRC. (**D**) The Kaplan-Meier plot of the prognostic signature based on the median of the risk score in the validation set. (**E**) The ROC curve for assessing the prediction value of the signature in the validation set.

**Figure 5:**
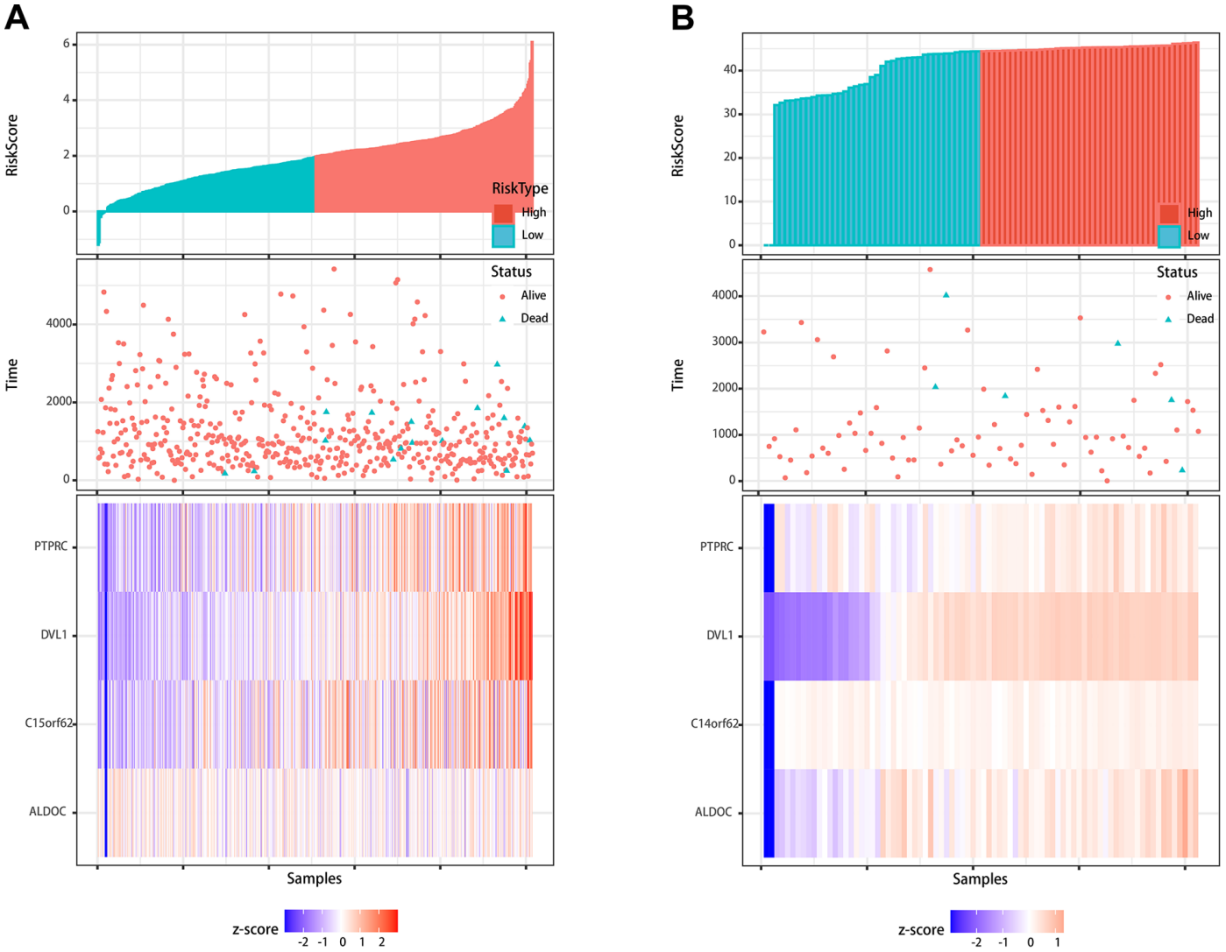
**Visualization of the prognostic signature.** (**A**) Visualization of the signature in the TCGA cohort. (**B**) Visualization of the signature in the validation cohort.

Furthermore, to evaluate the reliability and accuracy of the predictive model, we validated the power of the model in our own center. First, qMSP was performed to verify the expression of differentially methylated genes. As shown in Figure 4A, 4B, ALDOC, C14orf62, DVL1, PTPRC were hypermethylated, and their corresponding protein levels were decreased, which was consistent with the results from the TCGA cohort. Although there was no statistically significant difference between the methylation of C14orf62 and overall patient survival, the 4 MDGs joint model could successfully divide the TC patients into long-term OS group and short-term OS group, which indicated the effectiveness of the predictive model constructed based on 4 MDGs (Figure 4C, 4D). The AUC of the ROC curves of this model was 0.81 (5-year OS) (Figure 4E). Besides, as shown in Figure 5, we visualized the distribution of the 4 MDGs both on the training set and the validation set according to their risk scores by using R package ggrisk, suggesting that the higher the riskscore, the worse the prognosis. Also, the validation cohort was consistent with the TCGA cohort.

### Construction and validation of the nomogram

**Figure 6:**
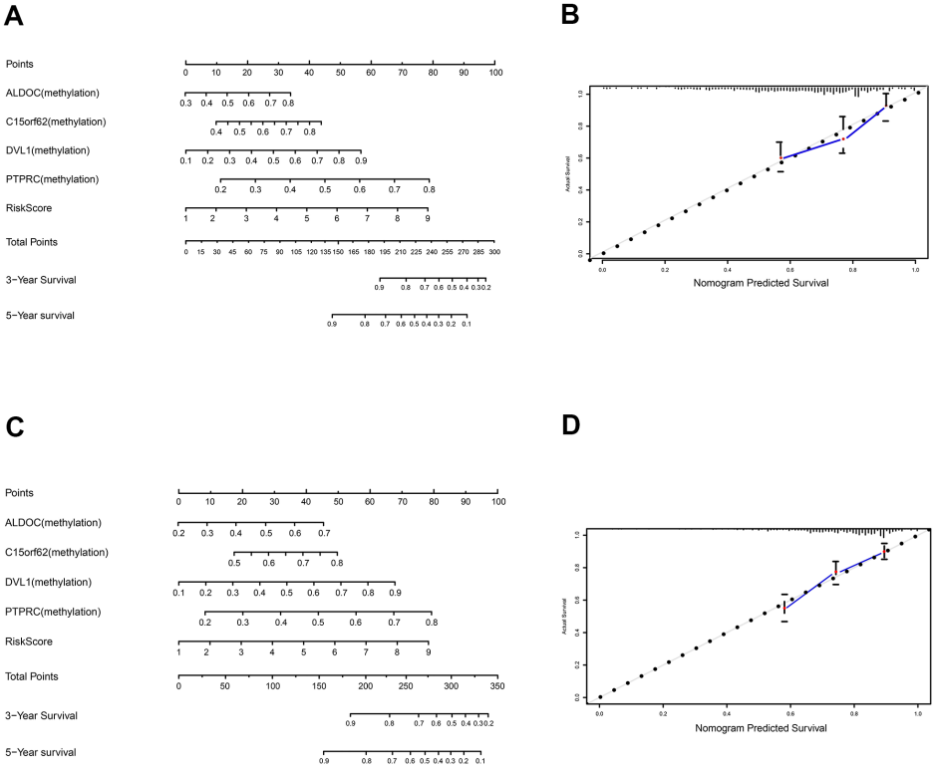
**Construction and validation of nomogram.** (**A**) The nomogram for predicting the survival with 3- and 5-year OS. (**B**) The calibration plot for validation of the model. (**C**) The nomogram for predicting the survival with 3- and 5-year OS in the validation set. (**D**) The calibration plot for validation of the model in the validation set.

To develop an effective method that could help predict an individual's prognostic risk of TC, we developed a nomogram based on the panel of these 4MDGs in the training set and validation set (Figure 6A, 6C). Calibration plots for predicting the 5-year survival rate showed that the nomograms performed well as compared with an ideal model both in the training set and validation set (Figure 6B, 6D).

### Knockdown of ALDOC, C14orf62, DVL1, and PTPRC significantly promoted the the proliferation, migration, and invasion of TC cells

**Figure 7:**
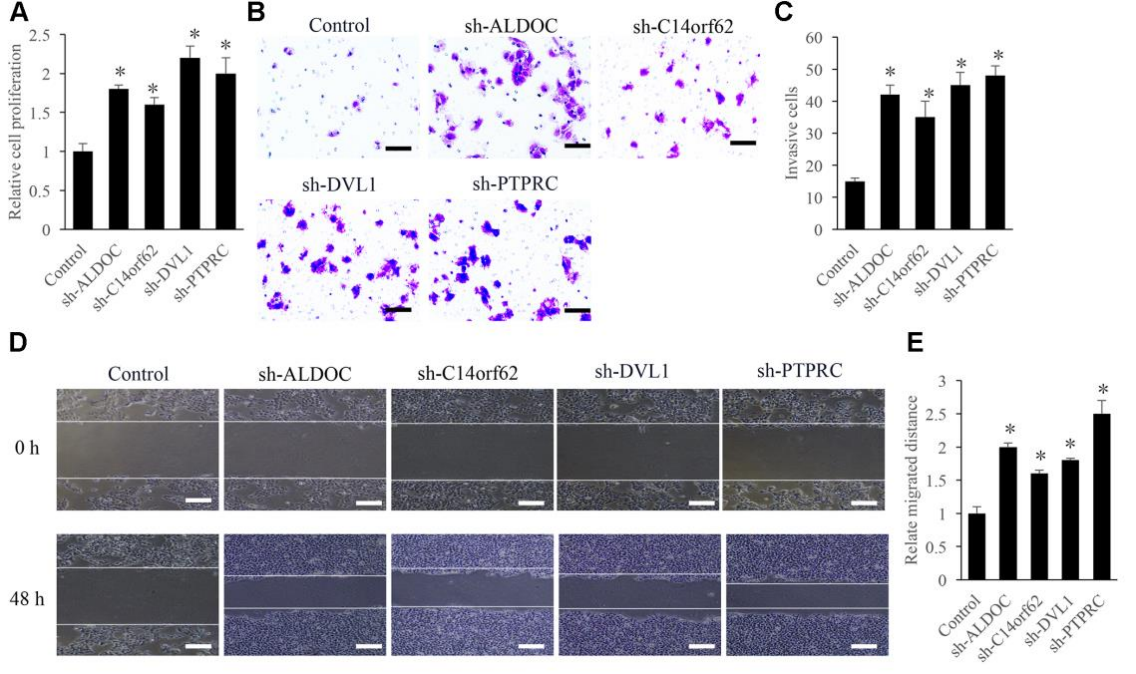
**Influence of ALDOC, C14orf62, DVL1, and PTPRC on the proliferation, migration, and invasion of TC cells.** (**A**) Influence of ALDOC, C14orf62, DVL1, and PTPRC on the proliferation of TC cells. (**B**) Influence of ALDOC, C14orf62, DVL1, and PTPRC on the invasion of TC cells (Scale bar=50 μm). (**C**) Quantitative analysis of the influence of ALDOC, C14orf62, DVL1, and PTPRC on the invasion of TC cells. (**D**) Influence of ALDOC, C14orf62, DVL1, and PTPRC on the migration of TC cells (Scale bar=500 μm). (**E**) Quantitative analysis of the influence of ALDOC, C14orf62, DVL1, and PTPRC on the migration of TC cells.

To investigate the regulation of ALDOC, C14orf62, DVL1, and PTPRC on the proliferation, migration, and invasion of TC cells, the knockdown vectors of ALDOC, C14orf62, DVL1, and PTPRC were constructed. We found that the cell proliferation ability was remarkably increased after treatment with sh-ALDOC, sh-C14orf62, sh-DVL1, and sh-PTPRC (Figure 7A). In addition, sh-ALDOC, sh-C14orf62, sh-DVL1, and sh-PTPRC significantly promoted the cell invasion (Figure 7B, 7C) and migration ability (Figure 7D, 7E).

## DISCUSSION

Thyroid cancer (TC) is one of the most commonly diagnosed cancers with a high rate of persistence and recurrence, and its incidence continues to be on the rise [22]. A variety of factors, including smoking, obesity, and radiation exposure, are considered as risk factors in TC. Despite the use of standard treatment methods, such as surgery or radioactive iodine, the prognosis of TC is still not completely elucidated. The mechanisms of TC remain unclear. Therefore, in-depth studies that screen for effective biomarkers are urgently needed for the early detection and treatment of TC. Recently, many studies have indicated that the over-expression of genes caused by hypomethylation and the low-expression of genes caused by hypermethylation play significant roles in the generation and progression of various tumors [23–25]. Aberrantly methylated genes may lead to gene expression disorders, transcriptional disorders, and abnormal cell differentiation [26, 27]. For example, Sugimoto et al. identified that the aberrant methylated GRWD1 gene may serve as a protective factor in cancer development. GRWD1 is an oncogene that can promote cell proliferation, and aberrant methylated GRWD1 inhibits its expression [28]. The aberrant methylation of other genes, such as FHIT [29], HOXA9 [30], NNK [31], and MSH3 [32] have also been found to be associated with cancer progression. With the use of databases such as GEO and TCGA, some information related to epigenetic and genetics can be studied. For instance, Lu and his colleagues investigated methylation-driven genes by using the R package methylmix with data from TCGA and found potential prognostic biomarkers and abberantly methylated sites associated with patient survival [33]. Also, Adib et al. used the framework to integrate multiple-cohort to identify methylation-driven subnetworks [34]. Therefore, the intersection between experiments and bioinformatics analysis is suitable for the screening of potential biomarkers for tumor patients.

In this study, in order to identify the MDGs in thyroid cancer, we used the R package methylmix to construct a mixed model to study the methylation levels of the genes. For this step, normalized methylation and gene expression data were used as the input matrix. Then, the 62 MDGs were found based on gene expression and corresponding methylation levels, while the correlation test was also performed using the Methylmix. Subsequently, functional analysis was used to study the potential mechanisms of these 62 MDGs. Among them, the results revealed that the MDGs were mainly enriched in substrate-dependent cell migration, cellular response to mechanical stimulus, cell-substrate adhesion, cellular response to tumor necrosis factor, dendritic cell differentiation. These findings not only suggest that aberrant methylation leads to cancer development, but also provides valuable information for understanding the mechanisms of TC development. Then, we turned our attention to the relationship between the 62 MDGs with prognosis. Clinical information was obtained through TCGA and was merged together with corresponding gene expression and methylation data. A set of 4-MDGs (ALDOC, C14orf62, DVL1, and PTPRC) based signature was constructed according to the univariate and multivariate COX regression analysis. It is noteworthy that although some of these genes were studied dysregulated in tumors, their methylation levels were rarely mentioned. For instance, ALDOC has been elucidated upregulated in gallbladder carcinoma (GBC) and reported to promote the growth of GBC by binding with MUC16 C-terminal [35]. ALDOC could directly activated transcription of the gene in melanoma cells [36]. EPB41 suppressed the Wnt/β-catenin signaling in non-small cell lung cancer by sponging ALDOC [37]. DVL1 might be involved in the early stages of astrocytoma malignancy [38]. Downregulated miR-1247-5p associated with poor prognosis and facilitates tumor cell growth via DVL1 in breast cancer [39]. DVL1 localized to CYP19A1 and regulated aromatase mRNA in breast cancer cells [40]. In addition, DVL1 was found to be associated with oxidative stress and may serve as an oxidation marker [41, 42]. However, there is no literature about the relationship between methylated DVL1 and TC. PTPRC, also known as CD45, GP180, has been extensively studied. As shown in Almanzar G et al. study, methylated PTPRC was participated in pro-inflammatory cytokine production, causing diffuse cutaneous systemic sclerosis [43]. Moreover, other studies have the aberrant methylation of PTPRC performing important functions in many biological processes [44, 45]. The reliability and stability of the proposed model were tested by performing qMSP and IHC using patient tissues from our center. These 4 MDGs were hypermethylated in the TC group, which consistent with the TCGA cohort. Additionally, the predictive model was also validated in our external verification, which provided a novel method for TC prognostic assessment.

Although the methylation signature was quite favorable, further reliability of the predictive model could be enhanced through the inclusion of more samples. The clinical risk factors, such as pathological subtype, gender, age, and staging, could be considered to integrate into the model. Further experiments and investigations are still needed to understand the mechanism of TC development and investigate effective approaches for the treatment of TC.

## CONCLUSIONS

In this study, we developed a favorable prognostic signature for TC based on the DNA methylation level of genes. The 4-MDGs (ALDOC, C14orf62, DVL1, and PTPRC) based prognostic model might be used to predict the survival rate of TC patients. This study provides a novel sight for accurate monitoring and prognosis assessment.

## MATERIALS AND METHODS

### Human tissues

The samples were collected from patients that underwent surgery at the department of thyroid surgery, the affiliated Hospital of Putian University. The study was approved by the Ethics Committee of Affiliated Hospital of Putian University (Approval number: 2019-036), and all patients or their guardians signed the consent form. Totally 82 TC tissues and adjacent normal tissues were used as a validation cohort. All tissues were immediately frozen in liquid nitrogen after the resection and stored at -80° C according to the manufacturer's protocol. The inclusion criteria is that the TC patients should be diagnosed via biopsy and histological testing. The exclusion criteria is that (1) Pregnant patients; (2) Significant immunodeficiency disease patients; (3) Patients with severe underlying diseases.

### DNA extraction, bisulfite modification and qMSP assay

Genomic DNA was extracted from the 82 TC tissues and paired normal tissues by using the TIANamp Genomic DNA Kit (#DP304, TIANGEN, Beijing, China). EZ Methylation-Direct Kit (#D502, Zymo Research, Irvine, CA, USA) was used for bisulfite conversion of 1000 ng from each sample according to the requirement of the instruction. The purified DNA was stored at −20° C. The purity/quality of DNA was measured using OD 260 nm/280 nm method, and the was OD 260 nm/280 nm value was 1.96 detected using NanoDrop 8000 (Thermo, Waltham, Massachusetts, USA).

qMSP was used to quantify methylation levels of the MDGs. The qMSP reaction consisted of a 1×ABI master mix containing Taq polymerase, dNTPs, 10 ng bisulfite-converted DNA, SYBR green dye and ROX as a passive dye (Thermo, Waltham, Massachusetts, USA) and 200 nM of specific primers. Specific primers for the promoter region of target genes were designed (Supplementary Table 1). Moreover, the ACTB gene, which did not contain any CpG dinucleotide, was used as a reference. The relative methylation level of each MDG was calculated using 2^(ΔCttarget- ΔCtreference)^.

### Data acquisition and pretreatment

The normalized mRNA expression and methylation data of TC were downloaded from the TCGA database. All data were transferred from the Genome Data Commons (GDC, https://gdc.cancer.gov/). The data includes mRNA expression from 568 specimens, including 510 tumor specimens and 58 normal specimens, as well as methylation level from 571 specimens, including 515 tumor specimens and 56 normal specimens. The R package edge was used to process the downloaded data to obtain levels of normalized expression and methylation. In addition, 507 samples with TC, which were accompanied by both clinical and expression information, were obtained from TCGA. The study was performed under the publication guidelines of TCGA and no restrictions were imposed in this research.

### Methylation-driven genes in TC

The methylmix is an efficient and accurate algorithm used for automatically analyzing the aberrantly methylated genes and the correlation between gene expression and methylation level. Before the application of the methylmix, preparation of documents for three datasets: cancer methylation data (METCancer), normal methylation data (METnormal), and matched gene expression data (GECancer), were normalized and obtained from the previous step. The 3 mentioned files were served as input files and then submitted to the methylmix according to the requirements of the algorithm. Briefly, three main steps were carried out: The first step was to identify the methylation status of genes by using the β-mixed model, which was applied for avoiding overfitting according to the Bayesian information criterion. Second, the Wilcoxon rank-sum test was performed to compare the methylation state between tumor and normal tissues to identify the significant difference on the basis of a Q-value of 0.05, which was performed using P-value multiple testing correction with false discovery rate (FDR). Finally, linear regression was used to model the expression of genes in terms of their DNA methylation. Considering the numbers of samples, **|**cor**|**> 0.3 was used as a standard criterion.

### Functional and pathway analysis

Furthermore, to explore the underlying mechanisms of these MDGs, gene ontology (GO) and Kyoto Encyclopedia of Genes and Genomes (KEGG) analysis were performed via the Metascape database (http://metascape.org). Additionally, the protein-protein interaction (PPI) network was used to identify the densely connected regions further. Here, P-value < 0.05 was used as a cutoff.

### Prognostic risk model construction

To further search for MDGs with prognostic value, Cox regression analysis was applied to assess univariate and multivariate associations of risk factors with the development of TC. Univariate Cox regression analysis was first performed to identify survival-associated MDGs. MDGs with a p-value < 0.05 were considered to be target genes. Multivariate Cox regression analysis was then applied to eliminate non-independent prognostic predictor genes. The prognostic model was established based on these target genes weighted by their estimated regression coefficients. Subsequently, Areceiver operating characteristic (ROC) was constructed to evaluate the predictive value of the prognostic model. The area under curve (AUC) was used to determine the efficiency of the model. The R package survival was applied in this section based on the Rstudio (Version 1.3.1073). Here, the data obtained from TCGA was used as a training set, and the 82 samples from our center were employed as the validation set.

### Construction and validation of the nomogram

The risk target genes obtained mentioned above were used for the nomogram model building to generate the predictive probability of 3-year and 5-year overall survival (OS). The C-index and calibration curves were applied to evaluate the internal validation of the nomogram. Besides, external validation of the predictive model was evaluated in a validation cohort. The R package rms was used to plot nomograms and calibration curvesbased on the Rstudio (Version 1.3.1073).

### Immunohistochemistry validation

Immunohistochemistry was performed according to the manufacturer's instruction. Sections were incubated with primary antibodies against aldolase C (AldoC), C14orf62, dishevelled 1 (DVL1), and protein tyrosine phosphatase receptor type C (PTPRC) overnight at 4° C. The image was captured at an appropriate magnification in the microscope (Nikon Microsystems, Shanghai, China).

### CCK8 assay

Cells were plated into the 96-well plate. After treatment with sh-ALDOC, sh-C14orf62, sh-DVL1, and sh-PTPRC for 48 h, the CCK-8 kit (#C0037, Beyotime, Beijing, China) was applied to detect cell proliferation. 20 μL regent was added to each well, OD at 450 nm was measured after 1 h incubation.

### Wound healing method

Cells were seeded into 6-well plates firstly. After reaching 70% confluence, 200 μL pipette tip was used to drawn a line in the middle plate. The medium was replaced with new medium. Then, the cells were incubated on the condition of 5% CO_2_ and 37° C. The cells were captured at 0 h and 48 h, and the relative migrated distance was analyzed.

### Transwell assay

The transfected cells were seeded into the upper chamber of a Transwell plate. The lower chambers were supplemented with 15% FBS (Gibco, Langley, OK, USA). After incubation (48 h), the cells were fixed using 70% ethanol (30 min). After staining with 0.2% crystal violet (10 min), the cells in the lower chamber were counted using a microscope.

### Ethics approval and consent to participate

The study was approved by the Ethics Committee of Affiliated Hospital of Putian University, and all patients or their guardians signed the consent form.

### Availability of data and materials

All data generated or analysed during this study are included in this published article.

## Supplementary Material

Supplementary Table 1
