# Supplementary material for: Identification of 4-methylation driven genes based prognostic signature in thyroid cancer: an integrative analysis based on the methylmix algorithm
Source: Aging (Albany NY). 2021 Aug 29;13(16):20164–78. doi: 10.18632/aging.203338 (PMC8436924; doi:10.18632/aging.203338)
Supplement: Supplementary Table 1 [file aging-13-203338-s001.pdf]

SUPPLEMENTARY TABLE

Supplementary Table 1. Primer sequence for the DNA methylation.

Gene	Methylated suquence
ALDOC	5′-TTTAGGTTCGGTATCGTTTCGC-3′<br>3'-CGAACTAAAAACGATACGCCG-5′
C14orf62	5′-GCCTAACGAAAAAAAATACGCG-3′<br>5'-ATTTTTTAGGTTTCGTTTCGGC-3'
DVL1	5′-AAAGATTCGGCGACCACCGAACGAC-3'<br>3′-GACTCAAACTCGAAAACTCGAA-5′
PTPRC	5′-CTGGTGCAGTATTTGATAGTGTA-3′<br>3′-TGAAAATGGTCAGAGAAACCTTTA-5′
ACTB	5′-GCTAAGTGTGCTGGGGTCTTGGGAT-3′<br>3′-GCTCTTTTTCTGGTGTTTGTCTCTC-5′
